# Effect of Thermal Processing and Heat Treatment Condition on 3D Printing PPS Properties

**DOI:** 10.3390/polym10080875

**Published:** 2018-08-06

**Authors:** Peng Geng, Ji Zhao, Wenzheng Wu, Yulei Wang, Bofan Wang, Shuobang Wang, Guiwei Li

**Affiliations:** School of Mechanical and Aerospace Engineering, Jilin University, Changchun 130025, China; gengpeng15@mails.jlu.edu.cn (P.G.); jzhao@jlu.edu.cn (J.Z.); yulei15@mails.jlu.edu.cn (Y.W.); wangbf1415@mails.jlu.edu.cn (B.W.); wangsb1416@mails.jlu.edu.cn (S.W.); ligw15@mails.jlu.edu.cn (G.L.)

**Keywords:** 3D printing, additive manufacturing, polyphenylene sulfide, thermal processing condition, heat treatment condition

## Abstract

Polyphenylene sulfide (PPS) is a high-performance semi-crystalline thermoplastic polymer that is widely used in the automotive, electronics, and aerospace industries, as well as other fields. However, PPS introduces several challenges in fused deposition modeling owing to its inherent properties of crystallization and thermal crosslinking. The present study demonstrates the effects of the thermal processing and heat treatment conditions on the accuracy and mechanical properties of PPS samples three-dimensionally printed through fused deposition modeling. By measuring the degree of crystallinity and thermal crosslinking of three-dimensionally printed PPS samples, we found that the thermal history affects the three-dimensionally printed PPS properties. Results show that the accuracy of three-dimensionally printed PPS samples can be improved by means of air-forced cooling in fused deposition modeling. The balance between mechanical strength and ductility was regulated by altering the heat treatment conditions. This approach is applicable to eliminating the warpage of semi-crystalline polymer in three-dimensional printing (not only for PPS) and provides a method of improving the mechanical properties of three-dimensionally printed PPS samples.

## 1. Introduction

Polyphenylene sulfide (PPS) is a semi-crystalline thermoplastic material having an asymmetrical rigid backbone chain comprising para-substituted phenylene rings and sulfur atoms. It is a polymer material having good dimensional stability, high-temperature stability, chemical resistance, and flame retardance, and it is easily processed. It also possesses the characteristics of aging resistance, radiation resistance, and nontoxicity [1,2,3,4,5]. PPS can be used in the electronics, automotive, and aerospace fields owing to its superior properties. The process of heating of PPS as a typical semi-crosslinking polymer obviously affects the final mechanical properties owing to the crystalline and cross-linking properties being dependent on the heat treatment history, ambient temperature, and other ambient conditions [6,7]. Park et al. [8] analyzed the solid-phase crosslinking process of PPS resin in the temperature range of 200–250 °C. Their results show that a higher oxygen concentration and ambient temperature increase the crosslinking rate and crosslinking degree of PPS.

Three-dimensional (3D) printing technology manufactures parts layer by layer from bottom to top [9]. 3D printing technology has surpassed traditional manufacturing methods in many respects: it provides more design freedom, is capable of quickly forming complex structural parts, and has the advantages of low manufacturing costs, a short development cycle, and high production efficiency [10]. Fused filament fabrication (FFF) is the most widely used technology in 3D printing. An object is deposited on a build platform by melting and extruding the polymer filament following the deposition trajectory, and 3D parts are ultimately built through accumulation layer by layer [11]. The materials commonly used with this technology are acrylonitrile butadiene styrene, polylactic acid, polycaprolactone and polycarbonate [12,13,14,15,16]. Most studies on 3D printing with PPS have focused on the exploration of printable parameters of PPS and its composites and the effect of printing parameters on warpage. Kishore et al. [17] formed high-performance semi-crystalline thermoplastic PPS and polyether ketone ketone and analyzed the rheological properties and thermal properties of these materials. On this basis, the parameters important to printability were determined. Fitzharris et al. [18] analyzed warpage due to residual stresses during the cooling of PPS and PP for different printing speeds through finite element analysis. DeNardo et al. [19] developed a CAMRI system for printing high-performance PPS with 50 wt. % carbon fibers. The system replaced the traditional molten extrusion system with a single screw extrusion system.

Complex thermal conditions affect the degree of crystallinity and the degree of crosslinking of the extruded material in the melting and deposition processes of a typical heat crosslinking semi-crystalline PPS. Controlling the thermal properties of the material in the forming process is important to improving the forming quality and accuracy of fused deposition modeling samples. Yang et al. [20] analyzed the effects of the ambient temperature and heat treatment conditions on the tensile properties of 3D-printed poly(ether ether ketone) (PEEK) samples. By controlling the thermal condition, PEEK samples with different elongations at break and tensile strengths were built. Wang et al. [21] analyzed the effects of the build platform temperature and layer thickness on the impact properties of 3D-printed polylactic acid samples. Their results showed that when the thickness of the layer was 0.2 mm and the substrate temperature was 160 °C, the 3D-printed samples had greater impact strength than injection molding samples. This greater impact strength is mainly due to the smaller cell sizes and lower molecular degradation compared with the injection sample. Kishore et al. [22] improved the surface temperature of the printing layer before the FFF process by means of infrared heating in a large-area 3D printing system to improve adhesion between the acrylonitrile butadiene styrene materials. The above studies reveal that the thermal condition in the process of FFF affects the mechanical properties of 3D-printed samples.

The present study therefore focuses on the thermal history of 3D-printed PPS samples and analyzes the effects of the thermal processing conditions and heat treatment condition on the mechanical properties (i.e., tensile strength, fracture resistance and impact strength) of the 3D-printed PPS samples. The degree of crystallinity, oxidation crosslinking and temperature distribution in the printing process under different thermal conditions were measured to reveal the relationships between the thermal history and the microstructure and mechanical properties of 3D-printed PPS samples. The mechanical properties of 3D-printed PPS samples can be controlled by adjusting the thermal treatment conditions.

## 2. Materials and Methods

### 2.1. Materials

Crosslinking PPS resin pellets (A900) were obtained from Toray Industries Co., Ltd. (Tokyo, Japan). The PPS filament with a diameter of 1.75 mm was extruded by a twin-screw extruder (sjzs-10z, Ruiming Experimental Instrument Co., Ltd., Wuhan, China). The temperatures of the three heating zones were 270, 289 and 280 °C. Before extrusion, PPS pellets were dried at 120 °C for 12 h to remove moisture.

### 2.2. Experimental Procedure

**Figure 1:**
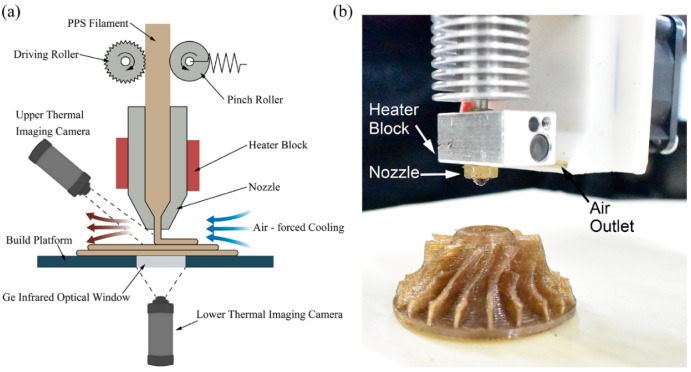
PPS 3D printing: (**a**) Schematic diagram of PPS 3D printing; (**b**) image of the extrusion head.

**Figure 2:**
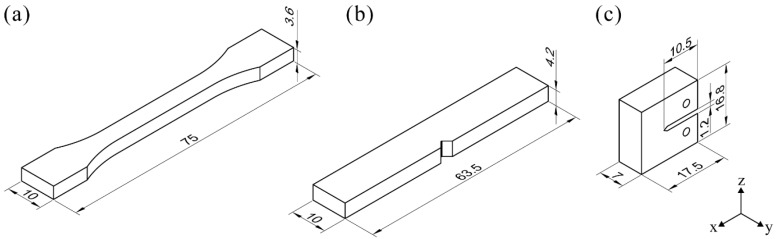
Dimensions for the 3D-printed PPS samples: (**a**) Tensile sample geometry; (**b**) Izod notched notched impact sample geometry; (**c**) compact tensile sample geometry.

A self-made PPS 3D printing system was adopted to print PPS samples. The 3D printing of PPS under an air-forced cooling condition and natural cooling condition was realized with a fan installed on the extrusion head. Figure 1 presents a schematic diagram of the PPS 3D printing and experimental equipment, while Table 1 gives the PPS 3D printing parameters. The temperature distribution of the upper/lower surface during printing was measured separately using an infrared thermograph (A310, FLIR System Inc, Boston, MA, USA). The lower surface temperature distribution was observed through germanium infrared optical windows, which were installed on the build platform of the 3D printer. All measurements were performed at 27 °C air temperature and a relative humidity of 60%. The distance between the camera lens and the sample’s surface was about 0.55 m. The emissivity coefficient of the 3D-printed samples was set at 0.91. The 3D-printed PPS models printing on the x–y plane with a layer thickness of 0.3 mm (z-direction) for mechanical performance testing are shown in Figure 2.

**Figure 3:**
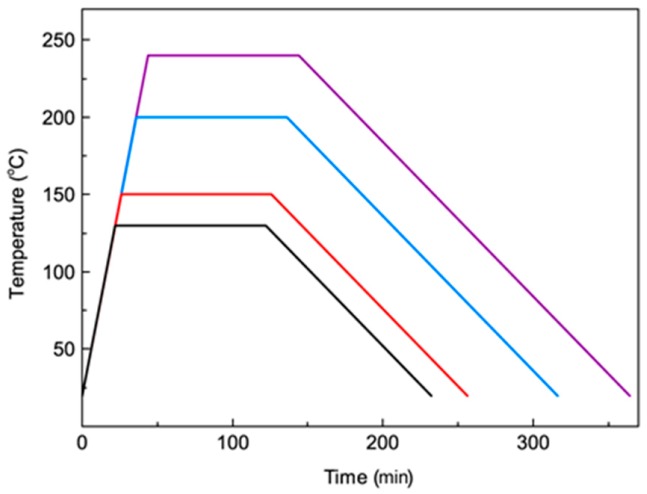
Heat treatment conditions of 3D-printed PPS samples.

To analyze the effects of heat treatment conditions on the properties of 3D-printed PPS samples, samples printed under the air-forced cooling condition were heated in air from room temperature to different heat treatment temperatures over a period of 100 min. The heat treatment conditions of 3D-printed PPS samples under air-forced cooling conditions are shown in Figure 3.

### 2.3. Testing of Mechanical Properties

Tensile tests, impact tests and Mode-I fracture tests were carried out according to ISO 527, ISO 180 and ISO 13586 respectively. The dimensions of test models were adjusted to ensure the uniformity of thickness of each layer after slicing the 3D model compared with the standard model. Tensile and fracture tests were carried out on an electronic universal testing machine (UTM5000, Shenzhen SUNS Technology Stock Co. Ltd., Shenzhen, China). The loading rate in the tensile test was 1 mm/min, while that in the fracture test was 10 mm/min. An Izod notched impact test was carried out on a Ceast pendulum impact tester (JJ-20, Changchun Intelligent Instrument and Equipment Co., Ltd., Changchun, China) with a pendulum energy of 5.5 J. Each value of a mechanical property is the average value of five samples under different thermal conditions. To improve the measurement accuracy of the dimensions of 3D-printed samples, a noncontact 3D scanner (EaScan-T, Hangzhou Shining 3D Tech Co., Ltd., Hangzhou, China) was employed to scan the 3D-printed samples and obtain the point cloud data of the printed sample. In Mode-I fracture tests, to ensure that the crack extended along the pre-notch in the loading process and reduce the residual stresses at the crack tip, a natural crack having a depth of 2 mm, as recommended in the test standard, was created during the printing process by adjusting the print layer thickness at the front end of the notch (from 0.3 to 0.2 mm). In the experiment, the curve of the force (*P*) versus displacement (*δ*) was recorded, and *P*_max_ and *P_Q_* were obtained according to the 5% secant line criterion of the force–displacement relation. The critical stress intensity factor *K*_IC_ was calculated as

(1) $$K_{Q}=\left(P_{Q}/BW^{1/2}\right)f\left(x\right),\;x=a/W$$

where 0.2 < *x* < 0.8, *a* is the crack length, *W* is the samples width, and

(2) $$f\left(x\right)=\;\frac{\left(2+x\right)\left(0.886+4.64x-13.32x^{2}+14.72x^{3}-5.6x^{4}\right)}{{\left(1-x\right)}^{\frac{3}{2}}}$$

### 2.4. Differential Scanning Calorimetry Analysis

A thermal analysis differential scanning calorimeter (Q2000, TA Instruments, New Castle, DE, USA) was used to determine the thermal response of 3D-printed PPS samples under different thermal conditions. All samples were heated from room temperature to 350 °C under a nitrogen atmosphere at a rate of 10 °C/min. To ensure the accuracy of crystallinity, 3–5 mg samples were cut from the inside of the fracture tensile samples. The degree of crystallinity (*X_c_*) under different thermal conditions was calculated as

(3) $$X_{c}=\left[\left(\Delta H_{m}+\Delta H_{c}\right)/\Delta H_{\theta }\right]\times 100\%$$

where Δ*H_c_* and Δ*H_m_* are respectively the enthalpies of recrystallization and melting. The melting enthalpy of 100% crystalline PPS (Δ*H_θ_*) is 77.5 J/g [23].

### 2.5. Fourier Transform Infrared Spectroscopy

Fourier transform infrared spectroscopy (FTIR) (Nicolet Nexus 670, Thermo Fisher Scientific, Waltham, MA, USA) was performed for the 3D-printed PPS samples under different thermal conditions with a single-reflection attenuated total reflectance (ATR) accessory. ATR-FTIR spectra were collected at room temperature over a scanning range of 4000–600 cm^−1^ with a spectral resolution of 4 cm^−1^. The surface of FTIR test is the cross sections of 3D-printed samples under different thermal conditions.

## 3. Results and Discussion

### 3.1. FTIR Analysis

**Figure 4:**
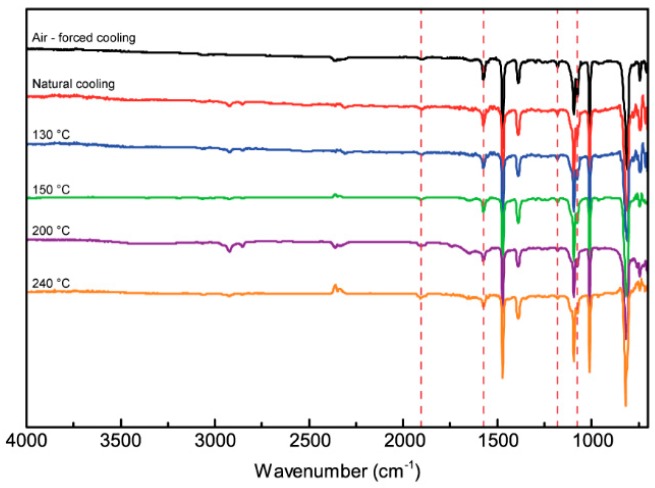
FTIR spectra of 3D-printed PPS samples.

Figure 4 shows the FTIR spectra recorded from 3D-printed PPS samples under different thermal conditions. All characteristic bands, including those of the benzene ring and C–H at 1571 and 1076 cm^−1^, are present. This indicates that PPS material maintains good stability after high-temperature treatment. After heat treatment, the PPS samples show obvious characteristic peaks at 1904 and 1180 cm^−1^ that should, respectively, be attributed to –C=O and –S=O stretching vibrations. This indicates that the peaks are formed by thermal oxidative crosslinking and an oxidation reaction in the printing PPS process. The thermal crosslinking of PPS depends on the temperature and time of exposure to air. In the printing process, although PPS was heated to its melting point, it cooled in just a few seconds. There was no obvious oxidative crosslinking phenomenon for the 3D-printed PPS samples.

### 3.2. DSC Analysis

**Figure 5:**
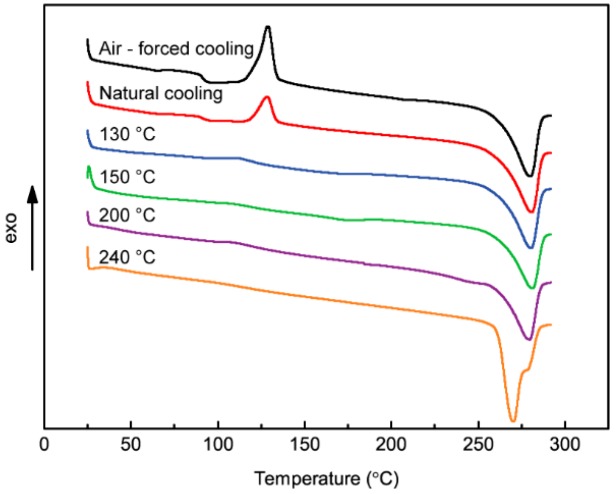
DSC curves of 3D-printed PPS samples.

Figure 5 shows crystallization and melting thermograms of 3D-printed PPS samples under different thermal conditions. Table 2 lists the quantitative data of the peak temperature and enthalpies. It is seen that the PPS crystallization temperature range is 126.37–135.27 °C. Furthermore, for both air-forced cooling and natural cooling conditions, there is an obvious recrystallization peak at 129 °C. When the samples are heated to 130–240 °C, the recrystallization peak diminishes or even disappears. This is mainly due to the sufficient reordering of the molecular chains in the sample, resulting in higher crystallinity and the establishment of a tighter polymer chain network. With an increase in the heat treatment temperature, the melting peak widens, which is mainly due to the thermal oxygen crosslinking reaction of the PPS molecules in the process of heat treatment and the greater number of C–O bonds. The PPS molecular chain undergoing oxidation and crosslinking has reduced molecular mobility during the cooling crystallization process that may not be sufficient to allow completion of the crystallization process. When the temperature increases again, the molecular chains with different molecular weights have slightly different mobility associated with a wider melting peak and the double melting peak.

### 3.3. Effect of the Thermal Processing Condition on Temperature Profiles and the Accuracy of 3D-Printed PPS Samples

**Figure 6:**
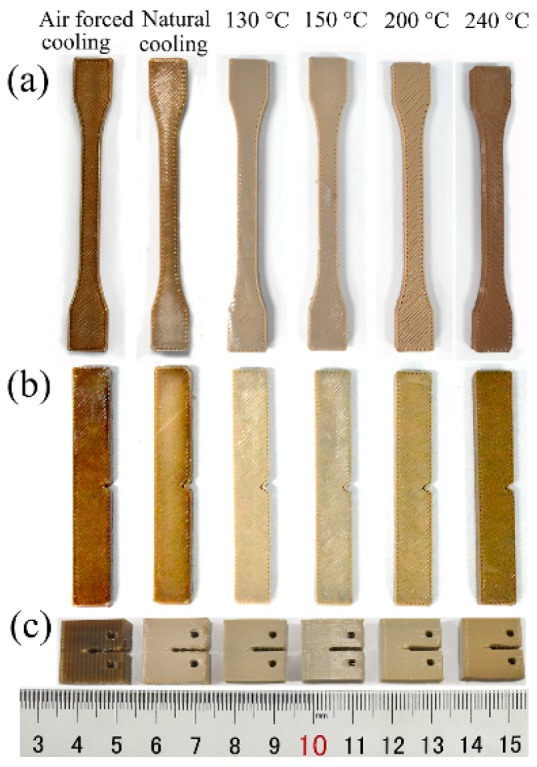
3D-printed PPS samples: (**a**) Tensile samples; (**b**) Izod notched impact samples; (**c**) compact tensile samples.

Figure 6 shows 3D-printed PPS samples. It is seen that the color of the PPS 3D-printed sample under the air-forced cooling condition is close to the color of the PPS pellet, while the color of heat-treated samples varies from ivory to brown in association with the increase in the heat treatment temperature.

**Figure 7:**
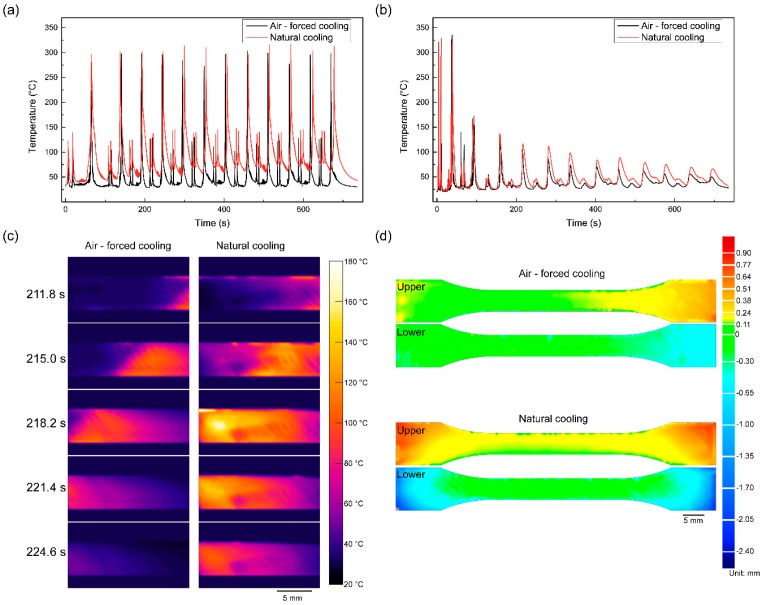
Evolution of the 3D-printed PPS temperature: (**a**) Temperature profile of the upper surface; (**b**) temperature profile of the lower surface; (**c**) infrared images showing the evolution of the lower surface temperature; (**d**) warpage of 3D-printed PPS samples.

Figure 7 shows the temperature profiles of the upper and lower surfaces of the 3D-printed PPS samples and the warpage of 3D-printed PPS samples. Figure 7a shows that the temperature at the sample center rises rapidly to the nozzle temperature and then decreases rapidly with by the nozzle leaving from measurement point. In the process of printing each layer, there are three peaks, specifically two secondary peaks that appear to coincide with the process of printing contours and a primary peak relating to the process of printing the infill pattern. Note that the primary peak reaches about 300 °C, which is different from the actual nozzle temperature of 285 °C. This difference is mainly due to the measurement being confounded by infrared reflections from the nozzle. In addition, emissivity was adopted by employing a semi-melted PPS material, while the thermal distribution was dominated by the brass nozzle when the nozzle moved past the measuring point, resulting in the deviation of the measurement. For the 3D-printed PPS samples under air-forced cooling, the peak temperature reduced rapidly and remained lower than *T*_c_. For the 3D-printed samples under the natural cooling condition, there was no adequate heat transfer in the envelope region. Figure 7b,c clearly shows that the heat-affected zone under the natural cooling condition is larger than that for the sample under the air-forced cooling condition. A comparison of the 3D-printed PPS samples and 3D model shows that the maximum deviation is +0.79/−0.89 mm under the air-forced cooling condition and +0.89/−2.38 mm under the natural cooling condition. It is seen that the sample under the condition of air-forced cooling is of high precision and the decrease of warpage due to residual thermal stress is attributed to the effective heat transfer through convection with the environment. In summary, the air-forced cooling method effectively controls the temperature distribution of the 3D-printed PPS sample and improves the accuracy of the 3D-printed PPS sample in the FFF process.

### 3.4. Tensile Properties of 3D-Printed PPS Samples

**Figure 8:**
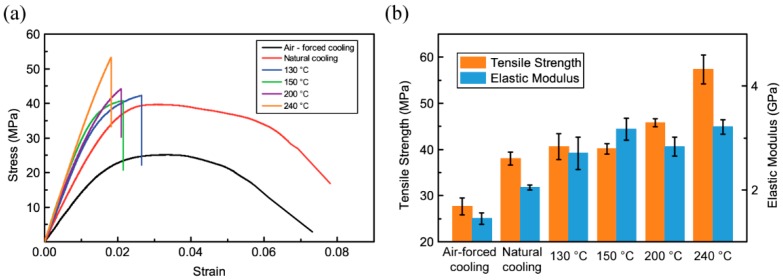
Tensile properties of 3D-printed PPS samples: (**a**) Stress–strain curves of the 3D-printed PPS samples; (**b**) elastic modulus and tensile strength of the 3D-printed PPS samples.

Figure 8 shows the tensile stress–strain curves and tensile strengths of 3D-printed PPS samples. It is seen that the heat treatment process affects the tensile properties of 3D-printed PPS samples. The 3D-printed PPS samples without heat treatment show greater break elongation. The 3D-printed PPS samples without heat treatment have a ductile behavior with plastic deformation. The samples show no obvious necking propagation and the fracture is at an angle of 45° to the loading direction. However, the samples that were heat treated had no obvious plastic deformation and necking propagation. As the loading increased, the samples broke rapidly after reaching maximum stress. These results indicate that a ductile-brittle transition in 3D-printed PPS samples occurred as the samples were heated above the crystallization temperature *T*_c_. Heat treatment is a feasible way to regulate the ratio of the crystalline region and amorphous region of 3D-printed PPS samples, and the ratio is a crucial factor affecting the mechanical properties. Figure 8b shows that the tensile strength of the sample at 240 °C was up to 108% higher than that of the sample subjected to air-forced cooling. The strengths and elastic moduli of 3D-printed samples increased with an increase in the heat treatment temperature.

The improvement in the tensile strength and elastic modulus is closely related to the crystallinity of materials and the crosslinking of molecular chains. Table 2 and Figure 6 reveal that with an increase in heat treatment temperature, the crystallinity rises from 19.13% to 64.08% and oxygen is continuously introduced in the molecular chain. Without considering the effects of the other printing parameters on the forming sample, the heat treatment method improves the tensile properties of 3D-printed PPS samples.

**Figure 9:**
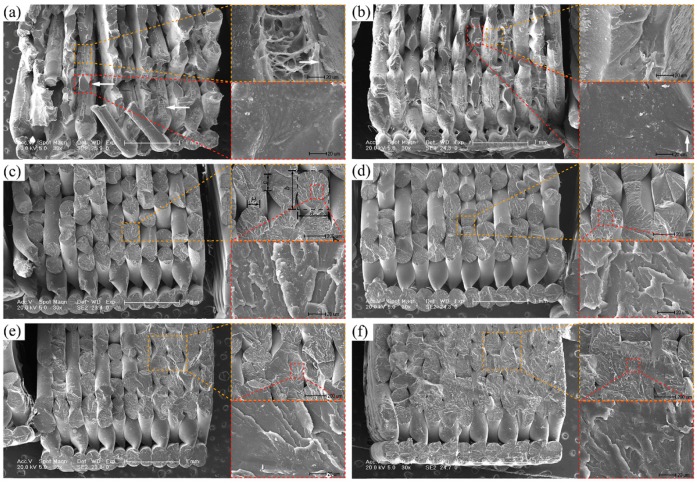
SEM micrographs of the fracture of 3D-printed PPS tensile samples under different thermal conditions: (**a**) air-forced cooling; (**b**) natural cooling; (**c**) 130 °C; (**d**) 150 °C; (**e**) 200 °C; (**f**) 240 °C.

It is worth noting that the mechanical properties of 3D-printed samples are not only related to the inherent properties of PPS materials but also depend on the macroscopic structure (i.e., voids between adjacent filaments, the directions of filaments and the diffusion degree at the interfaces of adjacent filaments) resulting from the complex printing trajectory. Figure 9 illustrates the fracture morphology of 3D-printed PPS samples. Figure 9a,b show localized necking and diffusion accompanying the increase in loading at the end of a filament, indicated by a white arrow in Figure 9a, and extensive crazing around the fracture. This is mainly because the semi-melted filament extruded from the nozzle produces a temperature gradient along the radial direction during solidification. The region close to the filament surface has sufficient heat convection with the environment, while the main cooling process at the core of a filament is heat transfer by conduction between adjacent filaments. The temperature gradient change results in a non-uniform crystallization region in which the outer filament is disordered and loose and the inner filament has stronger bonds among molecular chains. As the loading increases, the inner filament has better plastic deformation. When a critical value is reached, filament localized necking occurs, followed by micro-crack growth at the surface of the filament. Furthermore, diffusion between adjacent filaments results in a number of cavities and propagation associated with increasing loading due to insufficient bonding under the air-forced cooling condition. Figure 9b–f show that cavities are effectively suppressed when PPS samples are built at a higher environmental temperature.

Figure 9c,d show that the fracture contains a large number of flake-like structures and the necking phenomenon of the 3D-printed PPS sample disappears. The fracture surface roughness and the fracture extend in different directions at different levels [24]. With an increase in the heat treatment temperature, the void size reduces and the diffusion between the adjacent filaments and interlayer increases. When the heat treatment temperature reaches 240 °C, the fracture surface is perpendicular to the direction of the load, and the angle of 3D-printed samples heat treated at 130–200 °C between the fracture surface and the loading direction remains about 45°. The fracture morphologies of the 3D-printed PPS samples under heat treatment are covered with a fast-fracture zone and micro-ductile tearing, while the weak point of intercrystalline zones in the 3D-printed PPS samples under heat treatment at 240 °C is one reason for the fracture.

Table 3 gives the filament dimensions of 3D-printed PPS samples. The dimensions of a filament (a and b) remain constant, while the diffusion of the adjacent filament (*H*) and interlayer (*c*) increase. This is due to molecular motion being sufficient for the coalescence of cavities and the diffusion of interfaces when the heat treatment is above the glass transition temperature. The internal voids observed in Figure 9b are greatly reduced. It is therefore considered that the defects of the 3D-printed PPS samples can be effectively suppressed through heat treatment.

### 3.5. Fracture Toughness of 3D-Printed PPS Samples

**Figure 10:**
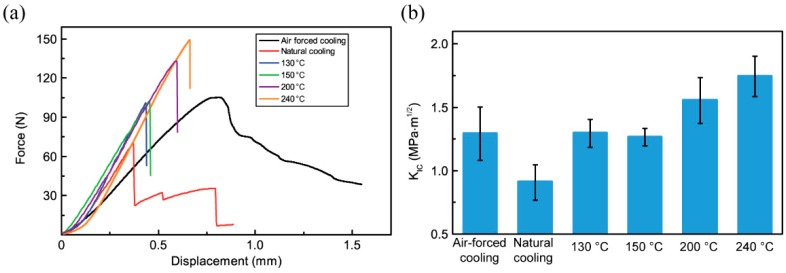
Fracture toughness of 3D-printed PPS compact tension samples: (**a**) The displacement and force curves; (**b**) the stress intensity factor *K*_IC_.

The fracture toughness of 3D-printed PPS samples is mainly attributed to interlayer bonding. Figure 10 shows the force–displacement curve and *K*_IC_. The sample had the best interlayer strength, followed by the sample heated at 240 °C. The sample that was naturally cooled had lower fracture toughness than the other samples. The samples under the natural cooling condition exhibit low interlayer strength. This may be caused by the residual stress leading to layer delamination and dislocation. Figure 8 and Figure 9 show that the samples that experienced air-forced cooling have typical plastic deformation behaviors. In fracture toughness tests, the samples under air-forced cooling were more able to resist crack extension, while the samples under heat treatment exhibited brittle fracture. Table 3 shows that the width of the interlayer bonding increases from 134.06 to 248.54 μm with the increase in heat treatment temperature. Heat treatment is an applicable method of improving the interlayer strength. The elimination of the interface between the semi-melt extrusion filament and the solidified previous filament provides resistance to fracturing. The heat treatment method therefore provides an effective way to improve the interlayer strength.

### 3.6. Izod Notched Impact Properties of 3D-Printed PPS Samples

**Figure 11:**
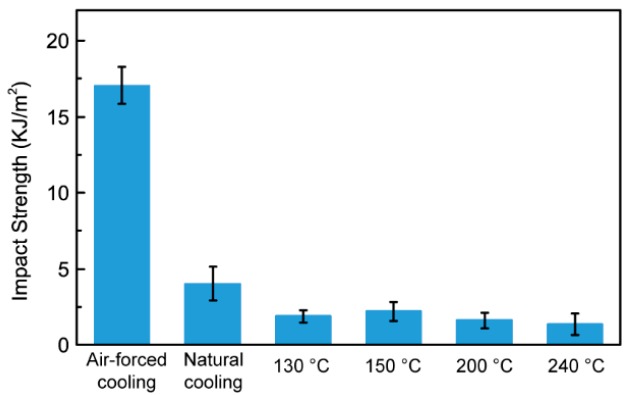
Izod notched impacted strength of 3D-printed PPS samples.

The impact strength is essentially an energy index that depends on the sum of various energies consumed by the material in the process of impact fracture. Figure 11 is the Izod notched impact strength of 3D-printed PPS samples. The figure shows that the impact strength of the PPS sample that experienced air cooling is 17.04 kJ/m^2^, which is obviously higher than the impact strength of the samples that underwent heat treatment. For the 3D-printed PPS sample, the impact properties of the sample are affected by the strength and modulus of the sample, the crystallinity and crosslinking of the material, the arrangement of the filaments, and the density of infill. The impact test mainly relates to the comprehensive state of the strength and plasticity of the material under the test conditions, and an increase in the plasticity of the material greatly improves the impact resistance of the material. When the sample is printed under air cooling conditions, the crystallinity of the sample is low and the amorphous region is larger. This leads to better ductility and greater toughness of the sample, and ultimately strong impact resistance. With the increase of heat treatment temperature, the samples exhibit a weaker resistance to the impact load and lower strength due to the increase of crystallinity.

## 4. Conclusions

It is important to analyze the effect of the thermal condition on the performance of 3D-printed PPS samples as PPS is a semi-crystalline thermoplastic polymer sensitive to oxidation. The PPS material undergoes a four-step (drying, screw extrusion, 3D printing and heat treatment) heating process from pellet to 3D-printed sample. The thermal history strongly affects the degree of crystallinity and crosslinking.

In the printing process, air-forced cooling improves the precision of the 3D-printed sample. The maximum deviation decreases from +0.89/−2.38 mm under the natural cooling condition to +0.79/−0.89 mm under the air-forced cooling condition. Most studies have focused on improving the environment temperature in an effort to restrain the non-uniform thermal gradients. We here propose reducing crystallinity by increasing the cooling rate to improve the precision of the 3D-printed sample.

The mechanical properties of 3D-printed samples are another important index. Heat treatment compensates for the insufficient strength of a sample that has undergone air cooling. A higher heat treatment temperature benefits the tensile strength and fracture toughness. The tensile strength increases from 27.7 to 57.3 MPa and the fracture toughness of 3D-printed PPS samples increases from 1.29 to 1.74 MPa·m^1/2^. Meanwhile, the elastic modulus increasing from 1.45 to 3.21 GPa, which is too high, results in the sample being unable to withstand a greater impact strength.

The mechanical properties of 3D-printed samples depend not only on inherent material properties (i.e., crystallization and crosslinking) but also on the 3D printing parameters (e.g., the infill and thermal processing conditions). The tensile strength and impact strength are mainly dictated by the degree of crystallinity and crosslinking. The heat treatment method provides an effective way to improve the interlayer strength. The diffusion of the interlayer, warpage and delamination depend on the thermal conditions during printing. The proper selection of thermal processing and heat treatment conditions will allow the processing of high-performance 3D-printed PPS samples.

## Figures and Tables

**Table 1:** Printing parameters of 3D-printed PPS.

Parameter	Value
Nozzle diameter	0.4 mm
Nozzle temperature	285 °C
Printing speed ^1^	680 mm/min
Layer thickness	0.3 mm
Number of contours	1
Infill pattern	Rectilinear
Raster angle	45°/−45°
Raster width	0.4 mm
Raster gap	0 mm
Extrusion multiplier	0.9
Outline overlap	0.06 mm

^1^ The first-layer printing speed was reduced by 50% to improve bed adhesion.

**Table 2:** Thermal properties of 3D-printed PPS samples.

Sample	Enthalpy (J/g)		Crystallinity (%)
	Δ*H_c_*	Δ*H_m_*	*X_c_*
Air-forced cooling	20.03	35.41	19.13
Natural cooling	9.31	36.77	35.43
130 °C	2.96	36.15	42.28
150 °C	--	33.31	42.75
200 °C	--	39.16	50.04
240 °C	--	49.67	64.08

**Table 3:** Measured filament dimensions of 3D-printed PPS samples.

Thermal Condition	H (μm)	a (μm)	b (μm)	c (μm)
130 °C	87.6 ± 8.2	290.7 ± 10.7	306.7 ± 15.6	134.1 ± 9.2
150 °C	108.8 ± 6.1	297.6 ± 12.1	380.8 ± 10.2	187.9 ± 10.5
200 °C	144.9 ± 10.5	299.4 ± 13.1	364.7 ± 8.9	240.8 ± 16.9
240 °C	204.9 ± 13.1	303.2 ± 11.2	370.8 ± 13.5	248.5 ± 19.8
